# Association between Blood Pressure Variability and frailty in Chronic Kidney Disease

**DOI:** 10.1093/geroni/igaf122.2364

**Published:** 2025-12-31

**Authors:** Danya Pradeep Kumar, Tobia Zanotto, Kate Young, Aditi Gupta

**Affiliations:** University of Kansas Medical Center, Kansas City, Kansas, United States; University of Kansas Medical Center, Kansas City, Kansas, United States; University of Kansas Medical Center, Kansas City, Kansas, United States; University of Kansas Medical Center, Kansas City, Kansas, United States

## Abstract

Blood pressure (BP) dysregulation is a common early sign of cardiovascular dysfunction and contributes to the progression of chronic kidney disease (CKD). BP variability (BPV), reflecting physiological BP fluctuations, is a marker of cardiovascular, physical, and cognitive aging. This single center cross-sectional study examines the association between frailty, and BPV and nocturnal BP dipping in CKD. We recorded 24-hour ambulatory blood pressure (ABP) in individuals with CKD. We assessed frailty using the FRAIL scale. BPV was examined for the 24-hour duration, daytime and nighttime hours (based on individual participant bedtimes). Nocturnal dipping for systolic and diastolic BP, and their association with frailty were examined. Twenty-eight individuals (46% female; age: 69.8±8.67 years; eGFR: 34.4±8.6 mL/min/1.73m2) with CKD completed the assessments. Based on the FRAIL scale, 10 participants were non-frail and 18 were pre-frail/frail. Nocturnal dipping was lower in the pre-frail/frail (systolic: 6.65±7.8%; diastolic: 11.2±8.7%) compared to the non-frail group (systolic: 13.1±9.0%; diastolic: 18.5±8.8%). Systolic BPV did not differ in the pre-frail/frail group (24-hour: 13.0±2.4%; day: 12.8±2.7%; night: 10.4±3.1%) compared to the non-frail group (24-hour: 14.4±2.8%; day: 12.9±2.4%; night: 11.2±3.5%). Lack of nocturnal BP dipping is associated with worse cardiovascular outcomes and higher mortality. Here we show that lack of nocturnal dipping is also associated with frailty. While cardiovascular causes and lack of nocturnal dipping could impact frailty, we cannot prove causality with this study. Addressing underlying factors for frailty and attenuation of nocturnal dipping, such as autonomic dysfunction, volume overload, and sleep disorders, may be important for improving outcomes in CKD.

